# Longitudinal Associations of Marital, Parenting, and Employment Transitions with Weight Gain in a Multi-Ethnic Asian Population Aged 21 Years and Above

**DOI:** 10.3390/ijerph18158115

**Published:** 2021-07-31

**Authors:** Clare Whitton, Yvonne Hui Min Wong, Rob M. van Dam

**Affiliations:** 1 Saw Swee Hock School of Public Health, National University of Singapore and National University Health System, Singapore 117549, Singapore; wong.yvonne@nus.edu.sg (Y.H.M.W.); rob.van.dam@nus.edu.sg (R.M.v.D.); 2 School of Public Health, Curtin University, Perth 6102, Australia; 3 Department of Nutrition, Harvard T.H. Chan School of Public Health, Boston, MA 02115, USA

**Keywords:** life span, weight gain, multi-ethnic, Asian

## Abstract

Identifying when most weight gain occurs throughout the life course can inform targeted public health interventions. We evaluated the association of childbirth, marriage, and employment changes with weight changes in a multi-ethnic Asian cohort. Singapore Multi-Ethnic Cohort participants ≥21 years (*n* = 9655) who identified as ethnic Chinese, Malay, or Indian were weighed and interviewed about marital status, employment, and number of children at baseline and after about four years. We used multivariable regression to evaluate life transitions in relation to weight change and major gain (≥5 kg), and adjusted for socio-demographic covariates. Weight gain was 3.55 kg (95% CI 3.17, 3.94) higher in young adults (21–30 years) compared with participants older than 60 years at baseline. Getting married was associated with weight gain in women, but not men (*p* interaction < 0.01). Women who got married gained 1.63 kg (95% CI 0.88, 2.38) more weight and were more likely to gain ≥5 kg (OR 1.99, 95% CI 1.35, 2.93) than those remaining unmarried. Having children was not associated with weight gain. Only among ethnic Indians, remaining a homemaker was associated with less weight gain than remaining employed. In this multi-ethnic Asian population, obesity prevention efforts should target young adulthood and, in women, the transition into marriage.

## 1. Introduction

The primary driver of the global rise in obesity is a shift in the distribution of body mass index of entire populations [1]. In most populations worldwide, adults tend to gain weight as they age [2]. Longitudinal evidence suggests the rate of weight gain fluctuates throughout the life course, and that patterns of weight gain differ by population and ethnic group [3,4]. Identifying when most weight gain occurs can inform public health interventions targeted at specific age groups or life transitions.

Transitions in both family life and employment status have been associated with weight gain. In a systematic review of studies conducted in western populations, in nine out of the twelve studies, entering marriage was associated with weight gain [5]. Similarly, becoming a parent has been associated with weight gain in US populations [6,7]. With regard to employment, working full-time was associated with greater weight gain compared with working part-time in Australian women [8]. In other studies in western populations, unemployment or job loss were associated with greater weight gain or a higher risk of obesity [9,10]. However, few studies have simultaneously examined these life transitions, making it difficult to ascertain their relative importance for weight gain trajectories during adulthood.

Most studies to date on life course weight gain have been conducted in western populations, and data for Asian populations are sparse [3,11]. It is therefore unclear whether the transitions associated with weight gain in western populations are also associated with weight gain in Asian populations. Studies of ethnically diverse samples have noted differences in life-transition-related weight gain by ethnic group [4,6,7]. For example, in the US, having more children was most strongly associated with being overweight in Hispanic men, while the number of children was not associated with BMI changes in black and white men and women [6]. In addition, in the US, white women had different patterns of age-related weight gain as compared with black women and black and white men [4]. Differences in socio-economic status and neighborhood environment can contribute to disparities in weight gain between racial and ethnic groups [12]. Parenting practices, education, language, and religion can be diverse in multi-ethnic societies, and may all impact health beliefs, attitudes, and behaviours. For example, cultural attitudes and traditional beliefs about foods have been shown to differ between Asian ethnic groups in Singapore [13] and may impact weight gain.

We therefore evaluated the association of age and common transitions during adulthood (age ≥ 21 years), including marriage, child birth, and changes in employment status, with changes in body weight in a multi-ethnic Asian cohort. The cohort includes participants of three major Asian ethnic groups: Chinese (East Asian), Indian (South Asian), and Malay (South East Asian). We hypothesized that the transitions associated with weight gain would reflect those identified in western populations, but that ethnic group differences may be present.

## 2. Materials and Methods

### 2.1. Participants and Study Design

The Multi-Ethnic Cohort (MEC) Study is an ongoing population-based cohort study of Singapore citizens and permanent residents aged 21 years and older, with several recruitment phases [14]. Two recruitment phases were included in this study. In phase 1, 14,465 participants were recruited at baseline, between 2004 and 2010. Sampling was stratified by ethnic group to have sufficient representation of three Asian ethnic groups: Chinese, Malay, and Indian. Details on the study methodology were previously published [14]. Of the phase 1 participants, 8340 were from several earlier cohort studies that were based on random samples of the Singapore population with disproportionate sampling to increase the numbers of ethnic Malays and Indians. In addition, 6125 participants were recruited through household visitations, referrals from existing cohort members, and recruitment drives at community events, mosques, and temples to enrich the number of ethnic Malays and Indians. In phase 2, 32,239 participants were recruited through household visitations at baseline, between 2013 and 2016.

MEC phase 1 participants were invited for a follow-up interview and health examination between 2013 and 2016. Of a total of 14,465 participants recruited at the phase 1 baseline, when approached to participate in a follow-up, 4011 declined to participate, 4022 were uncontactable, and 374 were ineligible. The phase 2 follow-up was still ongoing at the time of this study, and we included those who participated in the follow-up in 2018 and 2019. As of the end of 2019, 14,954 were interviewed, while 2143 declined to participate, 733 were uncontactable, and 668 were ineligible. Of these interviewed phase 2 participants, 11,726 underwent a health examination. Thus, a total of 17,815 MEC phase 1 and phase 2 participants were followed up, and their age, sex, and ethnicity were similar to those that were lost to follow-up (Table S1). Participants with a history of heart disease (*n* = 399), stroke (*n* = 80), cancer (*n* = 258), or diabetes (*n* = 2168), as determined by questionnaire, were excluded from analyses, as these conditions may have led to gain or loss of body weight. The total excluded due to disease history was 2887, as some participants had multiple disease conditions. As a result, a total of 9655 participants with complete information were used for the current analysis. Figure S1 details the exclusions. In MEC phase 2, females but not males were asked about number of children, therefore analyses on changes in number of children included 5435 participants. At baseline and follow-up, information on socio-demographic characteristics was collected through face-to-face interviews, and anthropometric measurements were performed during physical examinations at study sites. The study methodologies, protocols, and procedures were approved by the National University of Singapore Institutional Review Board (NUS IRB Ref: 12–140). Informed consent was obtained from all participants upon enrolment in the study.

### 2.2. Assessment of Exposure Variables and Covariates

Information on age, sex, ethnicity, highest level of education, and household income were collected using standardized questionnaires. The three lifestyle transitions investigated were changes in marital status, changes in job status, and changes in the number of children.

To assess marital status, the following question was asked at baseline and follow up: “What is your current marital status?” with the following options: “Never married”, “Currently married”, “Separated but not divorced”, “Divorced”, and “Widowed”. Because of smaller numbers, the categories “Divorced”, “Separated but not divorced”, and “Widowed” were merged. Transitions in marital status between categories baseline and follow-up were classified into the following: “Still not married”, “Still married”, and “Still divorced, separated, or widowed”, “Got married”, and “Got divorced, separated, or widowed”.

To assess occupational status, the following question was asked at baseline and follow up: “Which of the following best describes your usual work status over the last 12 months?” with the options: “Working”, “Homemaker/housewife”, “Retired”, “Student (full time)”, “Unemployed (able to work)”, “Unemployed (unable to work)”, and “Other”. Because of smaller numbers, the latter four categories were merged into “Student or unemployed”. Subsequently, transitions between baseline and follow-up were categorised into “Still working”, “Still homemaker”, “Still retired”, “Still student or unemployed”, “Got a job”, “Became homemaker”, “Became retired” and “Became a student or unemployed”.

To assess the number of children the participants had, “How many immediate family members do you have?” was asked at phase 1 baseline and “How many blood-related family members do you have?” was asked at phase 1 follow-up with the number of sons and daughters as one of the answer categories. The number of children at each time-point was calculated by adding the number of son(s) and number of daughter(s) indicated. Phase 2 female participants were asked “Did you give birth to any children?” and “How many children did you give birth to?” Life transitions related to the number of offspring were classified into the following four categories: “No children (No/No)” if participants indicated having no children at baseline and revisit, “Having first child (No/1st)” if they had indicated having no children at baseline and one or more at revisit, “Having more children (Yes/More)” if they already had at least one child prior to baseline and had more children at follow up, and “Same children (Yes/Same)” if they indicated they had children at baseline and the same number at revisit.

### 2.3. Outcome Measures

The main outcome of the study was the change in body weight with common lifestyle transitions. Participants had their weight in kilograms (kg) measured by SECA digital scales (SECA 700 series, Hamburg, Germany) and were instructed to remove items from their pockets before measurement at both baseline and follow-up. Subsequently the change in weight was calculated by the difference between measurement at baseline and follow-up. Only one follow-up measurement of body weight was obtained for each participant. A positive value for weight change indicated weight gain, and a negative value for weight change indicated weight loss. Height was measured on a portable stadiometer (SECA 200 series, Hamburg, Germany), after removing shoes, in centimetres at baseline. BMI was then calculated as weight divided by height squared (kg/m^2^). Categorisation of BMI was in accordance to the Asian BMI cut-offs [15]. We examined major weight gain as a secondary outcome and defined this as weight gain of 5 kg or more, as this has been associated with a substantially increased risk of obesity-related chronic diseases [16].

### 2.4. Statistical Analysis

We used multivariable linear regression to assess the association between life transitions (change in marital status, change in job status, and change in number of children) during follow-up and changes in body weight (kg) during the same period. In this model, we adjusted for sex (male, female), ethnicity (Chinese, Malay, Indian), height (cm), age at baseline (21–30, 31–40, 41–50, 51–60, and >60 years), highest level of education (<primary, primary, secondary, technical school, junior college/polytechnic, and university), and household income (<2000, 2000–3999, 4000–5999, 6000–9999, and >10,000 Singapore dollars, and ‘declined to answer’), and follow-up time. In addition, multivariable logistic regression was used to assess the odds of major weight gain associated with the various lifestyle transitions with adjustment for the same covariates. For our main analysis, we evaluated the association between different life transitions (i.e., marital status, having children, and employment status) in separate multivariable models. In additional analyses, we simultaneously included variables related to all these life transitions in the multivariable model. We tested interaction of life transitions with sex and ethnicity in relation to weight change by including multiplicative interaction terms in the multivariable models, and testing the significance of this addition with analysis of variance (ANOVA) F-tests. We also conducted analyses with stratification by sex and ethnicity.

Finally, splines were fitted by generating B-spline matrices with 6 degrees of freedom [17], and plotting weight change against age univariately. *p*-values < 0.05 were considered statistically significant. All statistical analyses were conducted using R Studio, Version 1.2.1335 [18], using the packages car [19], dplyr [20], ggplot2 [21], gridExtra [22], lubridate [23], and ggpubr [24].

## 3. Results

### 3.1. Baseline Characteristics

Characteristics of the study population are presented in Table 1. The average time between baseline and revisit was 3.9 years (IQR = 2.2). Of the participants, 57% were female, with an ethnic composition of 66% Chinese, 15% Malay, and 18% Indian. More than half (56.7%) of participants were overweight or obese at baseline. At baseline, the mean body weight was 65 kg (SD = 14), the mean height was 162.7 cm (SD = 8.8), and the mean BMI was 24.3 kg/m^2^ (SD = 4.5). The mean weight change from baseline to revisit was +1.42 kg (SD = 4.63) and 15.6% of participants experienced major weight gain (≥5 kg).

### 3.2. Baseline Socio-Demographic Characteristics and Weight Change

Table 2 shows associations between socio-demographic factors at baseline and weight gain during follow-up.

**Figure 1:**
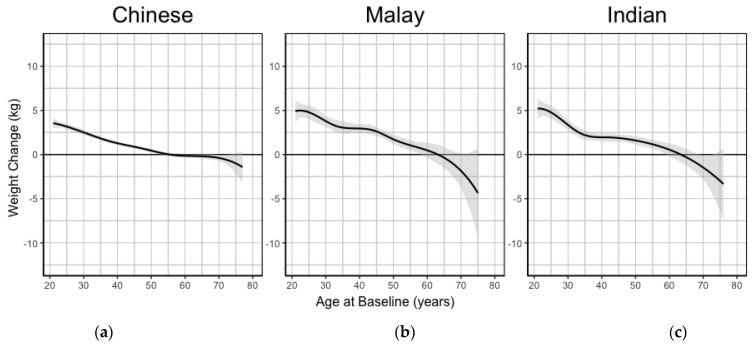
Weight change during follow-up according to baseline age in different ethnic groups among Singapore Multi-Ethnic Cohort participants aged 21 years and above. (**a**) Chinese participants; (**b**) Malay participants; (**c**) Indian participants.

Non-Chinese ethnicity, female sex, and younger age were significantly associated with greater weight gain and higher odds of major weight gain. In contrast, education and income levels were not significantly associated with weight gain. Younger baseline age was associated with greater weight gain, with a 3.55 kg (95% CI 3.17 to 3.94) greater weight gain for the youngest (21 to 30 years) as compared with the oldest (>60 years) group. Figure 1 displays a spline graph of the amount of weight gain during follow-up according to age at baseline. In all ethnic groups, weight gain was highest in young adulthood and slowed with increasing age, and weight loss occurred after about age 60 years.

### 3.3. Changes in Marital Status and Weight Gain

We examined changes in marital status in relation to change in weight and occurrence of major weight gain during follow-up (Table 3). Getting married was associated with significantly greater weight gain and a higher likelihood of major weight gain than remaining unmarried. In contrast, participants who were still married were less likely to have major weight gain than those that remained unmarried (OR 0.68, 95% CI 0.56 to 0.81). The association between getting married and weight gain differed significantly by sex (*p* interaction < 0.01), but not by ethnicity (*p* interaction = 0.10). Getting married was associated with weight gain in females (1.63 kg, 95% CI 0.88 to 2.38) but not males (0.40 kg, 95% CI −0.22, 1.02) (Table 4). Women who got married were also more likely to experience major weight gain (1.99, 95% CI 1.35, 2.93) than women who remained single. The association between getting married and weight gain in women was not substantially changed after adjustment for changes in having children and employment status (Table S2). Remaining divorced, separated, or widowed was associated with more weight gain in females (0.60 kg, 95% CI 0.03 to 1.17), but less weight gain in males (1.23 kg 95% CI 0.26 to 2.20), as compared with remaining unmarried (Table 4).

### 3.4. Changes in Number of Children and Weight Gain

Data on the number of children and weight gain were only available for female participants. Having a first child or having additional children was not significantly associated with weight gain (Table 3). Being a parent at baseline but not having further children during follow-up was associated with lower odds of major weight gain (OR 0.74, 95% CI 0.59 to 0.93) than having no children. No significant interaction was observed between ethnicity and changes in the number of children in relation to weight gain (*p* interaction = 0.25).

### 3.5. Changes in Work Status and Weight Gain

No associations were observed between changes in work status and weight gain in the overall study population (Table 3). The association between change in work status and weight gain differed significantly by ethnicity (*p* interaction < 0.01), but not by sex (*p* interaction = 0.26). An association between retiring and weight loss was observed in ethnic Indians (−2.92 kg, 95% CI −0.90 to −4.94) but not in ethnic Malay and Chinese (Table 5). In addition, in ethnic Indians, but not the other ethnic groups, remaining a homemaker was associated with weight loss (−1.21 kg, 95% CI −0.47 to −1.94) and lower odds of major weight gain (OR 0.44, 95% CI 0.29 to 0.67), as compared with remaining employed.

## 4. Discussion

In this longitudinal study, we evaluated the impact of various life course transitions on weight gain in a multi-ethnic Asian population aged 21 years and older. Younger age at baseline was a major determinant of greater weight gain, with the amount of weight gain gradually becoming less with older age and weight loss occurring after age 60 years. We also found that getting married was associated with greater weight gain and a higher likelihood of major weight gain in females. In contrast, having a first child or additional children was not significantly associated with weight change. Among employment transitions, we found that only among ethnic Indian participants, becoming retired or remaining a homemaker was associated with weight loss, as compared with remaining employed.

The strong association in our study between younger age and the amount of weight gained was consistent with findings of studies in U.S. [4,25,26,27,28] and Filipino [3] populations. This finding was consistent across Asian ethnic groups in our study, with similar patterns of weight gain according to age. In a multi-ethnic U.S. study of black and white populations, the pattern of 25-year weight gain differed by sex/ethnic group [4]. In that study, age-related attenuation of weight gain was observed in all groups except white women, whose weight gain did not slow with older age. However, this finding is in contrast to another US study in black and white populations in which a similar pattern of weight gain with age was observed across ethnic groups [26]. Our findings indicate the need for public health interventions to prevent excess weight gain in young adults in all Asian ethnic groups.

Two years of military service is mandatory for all 18-year-old male Singapore citizens and permanent residents, and this may contribute to weight gain in this age group, as observed in other settings such as the USA [29,30]. However, female participants do not go into military service and still experienced most weight gain between the ages of 21 and 30 years, suggesting that the relatively large weight gain in this age period is unlikely to be fully explained by military service.

We observed that the transition into marriage is associated with an average weight gain of about 1.6 kg and nearly 2-fold higher odds of major weight gain in women. This finding is consistent with results from a systematic review of 20 studies in US and European populations, in terms of the amount of weight gained in relation to the time span studied [5]. However, in contrast to these western studies, we did not observe a significant association between marriage and weight gain in Asian males. A US study explored the underlying mechanisms of weight gain following marriage, and reported evidence supporting two hypotheses [31]. In the ‘social obligation’ hypothesis, those in relationships eat more regular meals and larger portion sizes than single individuals because of obligations that arise as a result of the relationship. This hypothesis has been supported by other studies; for example, a US study found a significant association between change in participant BMI and change in spouse BMI over a 2-year period [32]. In the ‘marriage market’ hypothesis, unmarried individuals invest more effort in weight monitoring in order to attract a partner [31,33]. A U.S. study exploring the likelihood of becoming obese indicated that longer duration (>2 years) of a shared household environment with a romantic partner in young adulthood was associated with obesity, physical inactivity, and sedentary behavior [34]. In our study, remaining married was not associated with weight gain and was associated with a lower likelihood of major weight gain. Thus, the transition into marriage seems to be a key inflection point in weight gain in females in our population. Research on changes in dietary intakes and physical activity during the transition into marriage is warranted to understand the underlying causes of weight gain.

Remaining divorced, separated, or widowed in our study was associated with weight gain in women but weight loss in men. The lack of association between the transition of becoming divorced, separated, or widowed and weight change suggests that time is required for the impact of the change to become apparent. Changes in social support, depression, and stress may accompany a transition out of marriage and lead to changes in dietary intake and body weight [5]. In contrast to our findings, a review of marital transition and weight change reported that transition out of marriage was associated with weight loss in both men and women [5]. However, all studies in this review were in western populations. It is unclear why, for women in our study, remaining divorced, separated, or widowed was associated with weight gain, and this finding requires further investigation in other Asian populations.

Participants who were parents at baseline, and had no further children had lower odds of major weight gain, than participants with no children. Fertility rates in Singapore are among the lowest in the world [35], and vary by ethnic group. Overall, there are 1.1 births per female, with the highest fertility rate among females aged 30–34 years [36]. More than three-quarters of married females have children [36]; however, our analysis was able to separate the impact of getting married and having children. Some participants that got married did not have children, which may reflect the relatively short follow-up time and the tendency for there to be some years between getting married and having children. Much research on parenthood and weight gain has focussed on weight retention among postpartum women within the first months after childbearing [37,38]. However, a meta-analysis study which reported a decline in body weight within the first year postpartum [38] indicates that a longer follow-up time frame is needed to understand parental weight trajectories. Thus, early parenthood is not necessarily a key inflection point for weight gain in young adulthood, and weight gained immediately after having children may be lost later.

More than two-thirds of Singapore citizens and residents participate in the labor force [39], including older workers, who have an option for re-employment up to age 67 years, after reaching retirement age at 62 years [40]. In our study, retirement was associated with weight loss in ethnic Indians, independent of age. This association between retirement and weight loss is consistent with studies from European populations. In a Finnish cohort, retirement was associated with a BMI decrease in men. In that study, BMI decreases were largest in men with sedentary jobs [41]. In contrast, retirement was associated with weight gain in US women in blue-collar jobs, but not in men or white collar-workers [42]. We found no association between unemployment and greater weight gain, contrary to studies in northern European settings [9,10]. We found that remaining a homemaker was associated with less weight gain than remaining employed in ethnic Indians. This association has also been observed in an Australian female population [8], whereas in a UK cohort, long-term homemakers were more likely to be obese than women with some ties to the labor market [43]. Thus, the impact of changes in work status may differ in different settings and different population groups. This may reflect differences in the impact of these transitions on physical activity and dietary habits. For example, in Singapore, employed adults are more likely to eat out frequently than homemakers [44]. In contrast, in UK national survey data, working hours were not associated with the frequency of eating out [45].

Our study has several limitations. We did not capture the timing of the transition within the follow-up period. This may have attenuated some associations, if the impact of the transition changed over time. It may also have weakened our ability to detect short-term effects; however, the remaining longer-term effects are likely to be more relevant for obesity-related health outcomes. Loss to follow-up may also have affected the results. However, the socio-demographic profile of participants who participated in the revisit and those that were lost to follow-up was similar. The overall sociodemographic characteristics of our cohort reflect those of the general adult Singapore population, except for an over-sampling of ethnic Indians and Malays. It is therefore likely that our results are generalizable to the Singapore population, and possibly to other Asian populations, although this requires further investigation.

## 5. Conclusions

In conclusion, we observed that young adulthood is the period in which most weight gain occurred in ethnic Chinese, Malay, and Indian adults in Singapore. This is relevant as health screening programs and subsequent interventions often focus on children and middle-aged and older adults. Underlying mechanisms and contextual factors for weight gain in young adults should be examined to develop targeted interventions. Rather than only targeting individuals who are overweight or obese, such interventions should be population-wide, given the global shift in population distributions of body mass index [1].

Getting married, or remaining divorced, but not having children or gaining employment was a life transition that contributed to this weight gain in females. These findings may help to target public health interventions to prevent excess weight gain in the appropriate stages of adult life. When moving into marriage, adults are often supported by preparation courses, in Asian [46] and western settings [47], that may be a potential platform for interventions on healthy lifestyle practices.

## Figures and Tables

**Table 1:** Baseline characteristics of Singapore Multi-Ethnic Cohort participants aged 21 years and above.

Variables	Category	Total *N* = 9655	
Sex, *N* (%)	Male	4152	43.0%
	Female	5503	57.0%
Ethnicity, *N* (%)	Chinese	6411	66.4%
	Malay	1481	15.3%
	Indian	1763	18.3%
Age (years), *N* (%)	21–30	1396	14.5%
	31–40	2010	20.8%
	41–50	2982	30.9%
	51–60	2125	22.0%
	>60	1142	11.8%
Income level (S$), *N* (%)	<2000	1796	18.6%
	2000–3999	2419	25.1%
	4000–5999	1734	18.0%
	6000–9999	1348	14.0%
	>10,000	652	6.8%
	Declined to answer	1706	17.7%
Education level, *N* (%)	<Primary	568	5.9%
	Primary	985	10.2%
	Secondary	2221	23.0%
	Technical education	1503	15.6%
	Junior college/Polytechnic	2395	24.8%
	University	1983	20.5%
Body weight (kg), mean (sd)		64.5	13.8
Height (cm), mean (sd)		162.7	8.8
Body Mass Index, mean (sd)		24.3	4.5
Body Mass Index (BMI) Category, *N* (%) ^1^	Underweight (<18.5 kg/m^2^)	588	6.1%
	Normal (18.5–22.9 kg/m^2^)	3594	37.2%
	Overweight (23–27.5 kg/m^2^)	3230	33.5%
	Obese (>27.5 kg/m^2^)	2243	23.2%
Time to follow-up (years), median (IQR)		3.9	2.2

sd, standard deviation; IQR, interquartile range. ^1^ Categorization of BMI into categories in accordance with WHO Asian BMI Categorization.

**Table 2:** The association between socio-demographic characteristics and weight change during follow-up in Singapore Multi-Ethnic Cohort participants aged 21 years and above.

Characteristic		Outcome: Weight Change (kg) ^1^			Outcome: Major Weight Gain ^1^		
		Estimate (95% CI)		*p*-Value	OR (95% CI)		*p*-Value
Ethnic group	Chinese	0.00 (Ref.)			1.00 (Ref.)		
	Malay	1.23	(0.97, 1.50)	<0.01	2.35	(2.01, 2.75)	<0.01
	Indian	0.79	(0.55, 1.03)	<0.01	1.81	(1.57, 2.10)	<0.01
Sex	Male	0.00 (Ref.)			1.00 (Ref.)		
	Female	0.46	(0.20, 0.73)	0.001	1.22	(1.02, 1.45)	0.03
Age at baseline	21–30 years	0.00 (Ref.)			1.00 (Ref.)		
	31–40 years	−1.65	(−1.96, −1.35)	< 0.01	0.51	(0.43, 0.60)	<0.01
	41–50 years	−2.36	(−2.66, −2.07)	< 0.01	0.31	(0.26, 0.37)	<0.01
	50–60 years	−3.03	(−3.35, −2.70)	< 0.01	0.16	(0.13, 0.20)	<0.01
	>60 years	−3.55	(−3.94, −3.17)	< 0.01	0.08	(0.06, 0.12)	<0.01
Income at baseline	<SGD 2000	0.00 (Ref.)			1.00 (Ref.)		
	SGD 2000–3999	0.14	(−0.14, 0.41)	0.33	1.01	(0.84, 1.21)	0.92
	SGD 4000–5999	0.15	(−0.16, 0.46)	0.34	0.98	(0.80, 1.20)	0.85
	SGD 6000–9999	0.16	(−0.18, 0.50)	0.37	1.03	(0.82, 1.29)	0.80
	>SGD 10,000	−0.01	(−0.44, 0.42)	0.97	0.84	(0.61, 1.14)	0.26
Highest level of education attained	<Primary	0.00 (Ref.)			1.00 (Ref.)		
	Primary	−0.02	(−0.48, 0.45)	0.95	0.75	(0.53, 1.08)	0.12
	Secondary	0.17	(−0.25, 0.59)	0.43	0.76	(0.55, 1.05)	0.09
	Technical school	0.24	(−0.20, 0.68)	0.28	0.72	(0.52, 1.01)	0.06
	Junior college/Polytechnic	0.29	(−0.15, 0.73)	0.20	0.75	(0.54, 1.05)	0.09
	University	−0.25	(−0.71, 0.22)	0.30	0.52	(0.37, 0.74)	<0.01
Follow-up time (years)		0.21	(0.15, 0.27)	<0.01	1.16	(1.13, 1.20)	<0.01

OR, odds ratio; CI, confidence interval. ^1^ Models were additionally adjusted for baseline height.

**Table 3:** Longitudinal associations between lifestyle transitions and weight changes in Singapore Multi-Ethnic Cohort participants aged 21 years and above.

Model	Independent Variable	*N*	(%)	Outcome: Weight Change (kg) ^1^			Outcome: Major Weight Gain (≥5 kg) ^1^		
				Estimate	(95% CI)	*p*-Value	OR	(95% CI)	*p*-Value
Change in marital status	Still never married	1441	14.9%	0.00 (Ref.)			1.00 (Ref.)		
	Got married	425	4.4%	0.93	(0.45, 1.41)	<0.01	1.37	(1.07, 1.76)	0.01
	Still married	6971	72.2%	−0.22	(−0.52, 0.07)	0.14	0.68	(0.56, 0.81)	< 0.01
	Got divorced, separated, or widowed	276	2.9%	−0.52	(−1.12, 0.07)	0.08	0.83	(0.56, 1.22)	0.33
	Still divorced, separated, or widowed	541	5.6%	0.12	(−0.35, 0.60)	0.61	1.07	(0.77, 1.49)	0.68
Change in job status	Still working	6044	62.6%	0.00 (Ref.)			1.00 (Ref.)		
	Became homemaker	415	4.3%	0.28	(−0.18, 0.74)	0.23	1.04	(0.76, 1.41)	0.82
	Got a job	1021	10.6%	−0.08	(−0.39, 0.23)	0.61	0.93	(0.77, 1.12)	0.44
	Became retired	349	3.6%	−0.43	(−0.94, 0.07)	0.09	1.09	(0.69, 1.74)	0.70
	Became student	43	0.4%	−0.71	(−2.05, 0.63)	0.30	0.70	(0.34, 1.42)	0.32
	Still homemaker	1154	12.0%	−0.23	(−0.54, 0.07)	0.14	0.81	(0.65, 1.01)	0.06
	Still retired	307	3.2%	−0.07	(−0.65, 0.51)	0.81	1.01	(0.52, 1.94)	0.98
	Still student	28	0.3%	−1.06	(−2.72, 0.60)	0.21	0.57	(0.22, 1.43)	0.23
	Became unemployed	246	2.5%	−0.23	(−0.80, 0.34)	0.43	0.95	(0.65, 1.39)	0.81
	Still unemployed	48	0.5%	−0.04	(−1.30, 1.22)	0.95	0.85	(0.34, 2.12)	0.72
Change in number of children ^2^	No children	1157	21.3%	0.00 (Ref.)			1.00 (Ref.)		
	Had 1st child	338	6.2%	0.42	(−0.16, 1.00)	0.15	0.97	(0.69, 1.34)	0.83
	Had additional child(ren)	734	13.5%	−0.03	(−0.50, 0.43)	0.88	0.86	(0.66, 1.13)	0.29
	Parent, but no additional child	3206	59.0%	−0.08	(−0.42, 0.27)	0.67	0.74	(0.59, 0.93)	0.01

OR, odds ratio; CI, confidence interval. ^1^ All effect estimates were adjusted for ethnicity, sex, height, age at baseline, income at baseline, education at baseline, and time to follow-up. ^2^ Model on change in number of children was conducted on subset of main dataset.

**Table 4:** Associations between changes in marital status and weight change stratified by sex in Singapore Multi-Ethnic Cohort participants aged 21 years and above.

Model	Independent Variable	N	(%)	Outcome: Weight Change (kg) ^1^			Outcome: Major Weight Gain (≥5 kg) ^1^		
				Estimate	(95% CI)	*p*-Value	OR	95% CI	*p*-Value
Male	Still not married	659	15.9%	0.00 (Ref.)			1.00 (Ref.)		
	Got married	248	6.0%	0.40	(−0.22, 1.02)	0.21	1.10	(0.79, 1.53)	0.56
	Still married	3090	74.4%	−0.39	(−0.85, 0.08)	0.10	0.68	(0.51, 0.90)	0.01
	Got divorced, separated, or widowed	63	1.5%	−0.15	(−1.27, 0.97)	0.79	0.62	(0.28, 1.35)	0.23
	Still divorced, separated, or widowed	92	2.2%	−1.23	(−2.20, −0.26)	0.01	0.65	(0.28, 1.51)	0.31
Female	Still not married	782	14.2%	0.00 (Ref.)			1.00 (Ref.)		
	Got married	178	3.2%	1.63	(0.88, 2.38)	<0.01	1.99	(1.35, 2.93)	<0.01
	Still married	3881	70.5%	0.03	(−0.36, 0.42)	0.88	0.75	(0.58, 0.96)	0.02
	Got divorced, separated, or widowed	213	3.9%	−0.47	(−1.19, 0.24)	0.20	0.95	(0.60, 1.51)	0.83
	Still divorced, separated, or widowed	449	8.2%	0.60	(0.03, 1.17)	0.04	1.26	(0.85, 1.86)	0.24

OR, odds ratio; CI, confidence interval. ^1^ All effect estimates were adjusted for ethnicity, height, age at baseline, income at baseline, education at baseline, and time to follow-up.

**Table 5:** Associations between changes in employment status and weight change stratified by ethnicity in Singapore Multi-Ethnic Cohort participants aged 21 years and above.

Model	Independent Variable ^2^	N	(%)	Outcome: Weight Change (kg) ^1^			Outcome: Major Weight Gain (≥5 kg) ^1^		
				Estimate	(95% CI)	*p*-Value	OR	(95% CI)	*p*-Value
Chinese	Still working	4068	63.5%	0.00 (Ref.)			1.00 (Ref.)		
	Got a job	668	10.4%	0.24	(−0.10, 0.58)	0.17	1.08	(0.84, 1.39)	0.54
	Still homemaker	609	9.5%	−0.06	(−0.43, 0.31)	0.74	0.92	(0.64, 1.33)	0.66
	Became homemaker	255	4.0%	0.08	(−0.44, 0.60)	0.77	0.75	(0.43, 1.32)	0.32
	Still retired	274	4.3%	0.04	(−0.52, 0.61)	0.88	0.98	(0.45, 2.11)	0.96
	Became retired	299	4.7%	−0.19	(−0.70, 0.31)	0.45	1.43	(0.85, 2.42)	0.18
	Became unemployed	156	2.4%	−0.20	(−0.85, 0.44)	0.54	0.86	(0.49, 1.52)	0.61
Malay	Still working	872	58.9%	0.00 (Ref.)			1.00 (Ref.)		
	Got a job	168	11.3%	−0.76	(−1.70, 0.18)	0.11	0.61	(0.40, 0.92)	0.02
	Still homemaker	272	18.4%	0.12	(−0.74, 0.98)	0.79	0.93	(0.64, 1.36)	0.71
	Became homemaker	77	5.2%	0.34	(−0.98, 1.66)	0.61	1.00	(0.57, 1.78)	0.99
	Still retired	16	1.1%	−2.51	(−5.45, 0.43)	0.09	0.57	(0.06, 5.05)	0.61
	Became retired	24	1.6%	−0.83	(−3.08, 1.42)	0.47	1.04	(0.33, 3.29)	0.95
	Became unemployed	39	2.6%	−1.67	(−3.41, 0.08)	0.06	0.65	(0.28, 1.49)	0.31
Indian	Still working	1104	62.6%	0.00 (Ref.)			1.00 (Ref.)		
	Got a job	185	10.5%	−0.68	(−1.48, 0.13)	0.10	0.83	(0.57, 1.23)	0.36
	Still homemaker	273	15.5%	−1.21	(−1.94, −0.47)	<0.01	0.44	(0.29, 0.67)	<0.01
	Became homemaker	83	4.7%	0.78	(−0.37, 1.93)	0.18	1.24	(0.72, 2.13)	0.45
	Still retired	17	1.0%	−1.03	(−3.57, 1.51)	0.43	1.58	(0.30, 8.30)	0.59
	Became retired	26	1.5%	−2.92	(−4.94, −0.90)	<0.01	N.A. ^3^	-	-
	Became unemployed	51	2.9%	0.46	(−0.94, 1.85)	0.52	1.25	(0.65, 2.44)	0.50

OR, odds ratio; CI, confidence interval. ^1^ All effect estimates were adjusted for sex, height, age at baseline, income at baseline, education at baseline, and time to follow-up. ^2^ Results on becoming a student, still a student, and still unemployed are not shown because of low numbers. ^3^ No ethnic Indians who became retired experienced major weight gain.

## Data Availability

The data that support the findings of this study are available on request from the Saw Swee Hock School of Public Health data request team (http://blog.nus.edu.sg/sphs/, accessed on 29 July 2021).

## Supplementary Materials

The following are available online at https://www.mdpi.com/article/10.3390/ijerph18158115/s1, Figure S1: Exclusion flow chart, Table S1: Demographics of Singapore Multi-Ethnic Cohort participant dataset and those lost to follow-up, Table S2: Associations between changes in marital status and weight change stratified by sex.
