# Supplementary material for: Longitudinal Associations of Marital, Parenting, and Employment Transitions with Weight Gain in a Multi-Ethnic Asian Population Aged 21 Years and Above
Source: Int J Environ Res Public Health. 2021 Jul 31;18(15):8115. doi: 10.3390/ijerph18158115 (PMC8345590; doi:10.3390/ijerph18158115)
Supplement: Supplementary file 1 [file ijerph-18-08115-s001.zip › ijerph-1264391-supplementary.pdf]

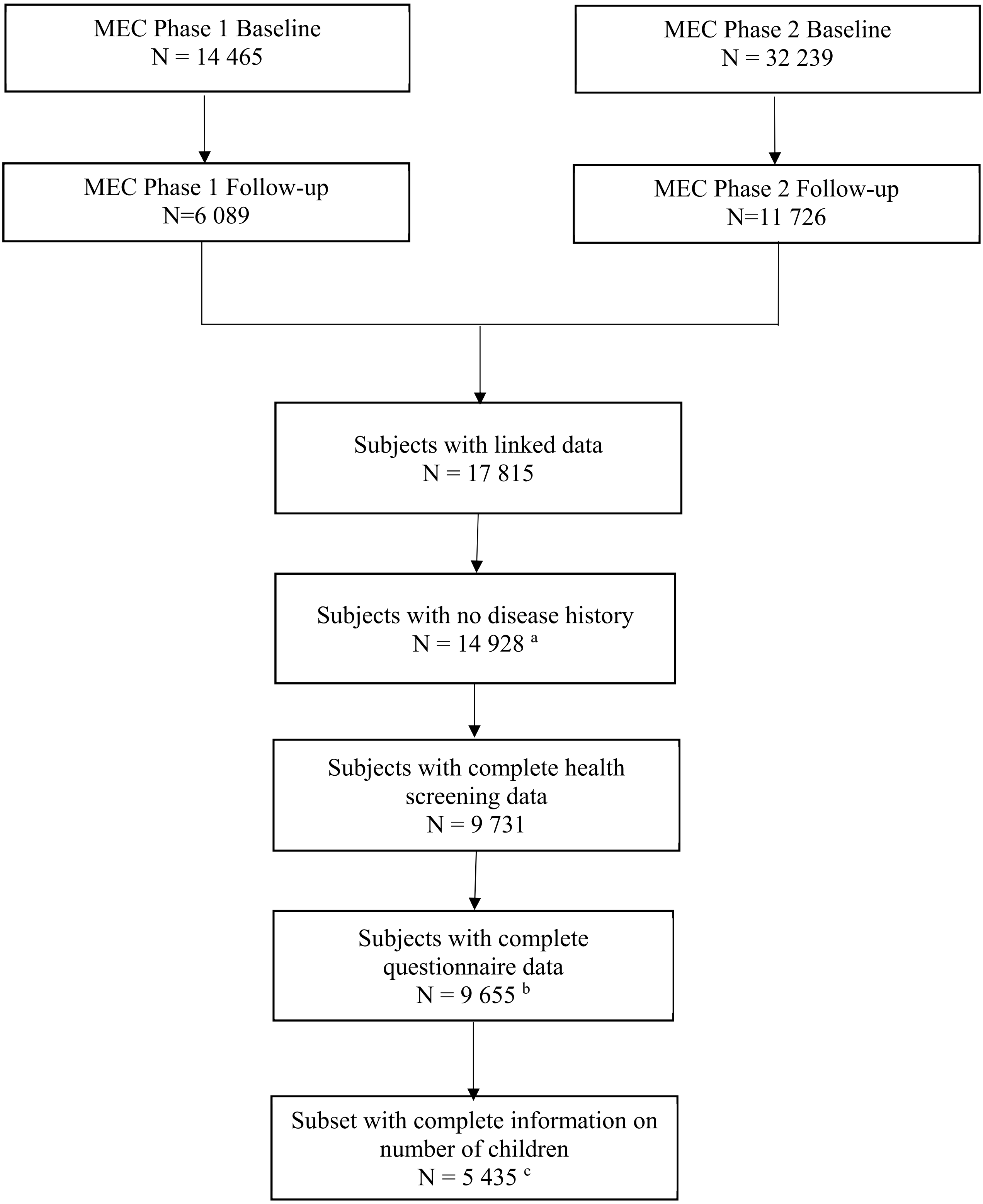

Figure S1: Exclusion flow chart. MEC, Multi-Ethnic Cohort. [a] history of heart disease, cancer, stroke, or diabetes. [b] Dataset used for analysis in project except otherwise stated.[c] Dataset used for analysis concerning offspring information. During the first part of MEC baseline respondents were only asked total number of family members and as such, the number of children was not recorded. In MEC phase 2 follow-up, only females were asked about number of children. Thus, the n = 5 435 are all female.

Table S1: Demographics of Singapore Multi-Ethnic Cohort participant dataset and those lost to follow up

Characteristic	Category	Lost to follow up N = 28973		Dataset before exclusions N = 17815		P-value
Sex, N (%)	Male	12757	44.0%	7658	43.0%	0.02 [a]
	Female	16179	55.8%	10150	57.0%	
Ethnic group, N (%)	Chinese	17282	59.6%	11097	62.3%	< 0.01 [b]
	Malay	5763	19.9%	2930	16.4%	
	Indian	5837	20.1%	3765	21.1%	
	Others	85	0.3%	23	0.1%	
Age, mean (sd)		46.07 (14.3)		46.25 (13.2)		0.17 [c]

sd, standard deviation

[a] Test of difference of proportions

[b] Chi-square test of difference

[c] T-test of difference of means

Table S2: Mutually adjusted associations between changes in employment, marital status, and having children, and weight change during follow-up in women[a].

Variable	N	(%)	Outcome: Weight change (kg)				Outcome: Major weight gain (≥ 5 kg)			
			Estimate[b]	*(95% CI)*	*p-value*	*Overall p*	*OR*[b]	*(95% CI)*	*p-value*	*Overall p*
Employment										
Still working	2774	51.0%	0.00 (Ref.)			< 0.01	1.00 (Ref.)			< 0.01
Became homemaker	401	7.4%	0.27	(-0.22, 0.76)	0.28		0.93	(0.67, 1.29)	0.66	
Got a job	697	12.8%	-0.15	(-0.54, 0.24)	0.44		0.84	(0.66, 1.07)	0.16	
Became retired	170	3.1%	-0.07	(-0.82, 0.67)	0.85		0.82	(0.40, 1.70)	0.60	
Became student	13	0.2%	-0.21	(-2.70, 2.29)	0.87		0.49	(0.10, 2.41)	0.38	
Still homemaker	1141	21.0%	-0.22	(-0.56, 0.12)	0.21		0.78	(0.62, 0.99)	0.04	
Still retired	89	1.6%	0.58	(-0.46, 1.61)	0.27		1.45	(0.54, 3.94)	0.46	
Still student	14	0.3%	-0.56	(-3.00, 1.87)	0.65		0.77	(0.20, 2.95)	0.71	
Became unemployed	117	2.2%	-0.16	(-1.02, 0.69)	0.71		1.18	(0.71, 1.96)	0.53	
Still unemployed	19	0.3%	0.43	(-1.65, 2.51)	0.69		1.11	(0.31, 3.95)	0.87	
Marriage										
Still never married	782	14.4%	0.00 (Ref.)			< 0.01	1.00 (Ref.)			< 0.01
Got married	176	3.2%	1.57	(0.76, 2.37)	< 0.01		2.04	(1.34, 3.10)	< 0.01	
Still married	3830	70.5%	0.10	(-0.50, 0.70)	0.74		0.84	(0.58, 1.21)	0.34	
Got divorced, separated, or widowed	209	3.8%	-0.43	(-1.27, 0.42)	0.32		1.05	(0.62, 1.79)	0.84	
Still divorced, separated, or widowed	438	8.1%	0.64	(-0.08, 1.36)	0.08		1.34	(0.84, 2.13)	0.22	
Having children										
No children	1157	21.3%	0.00 (Ref.)			< 0.01	1.00 (Ref.)			< 0.01
Had 1st child	338	6.2%	0.30	(-0.39, 0.98)	0.40		1.02	(0.68, 1.51)	0.94	
Had additional child(ren)	734	13.5%	-0.03	(-0.63, 0.56)	0.91		1.04	(0.73, 1.48)	0.83	
Parent, but no additional child	3206	59.0%	-0.08	(-0.60, 0.43)	0.76		0.87	(0.63, 1.20)	0.39	

OR, Odds Ratio; CI Confidence Interval

[a] Results are only shown for women, because data on having children were not available for men. [b] All effect estimates were adjusted for ethnicity, height, age at baseline, income at baseline, education at baseline and time to follow-up
